# Mass Transfer of Proteins in Aqueous Two-Phase Systems

**DOI:** 10.1038/s41598-019-39797-9

**Published:** 2019-03-06

**Authors:** I. Kaplanow, F. Goerzgen, J. Merz, G. Schembecker

**Affiliations:** Laboratory for Plant and Process Design, Department of Biochemical and Chemical Engineering, TU Dortmund University, D-44227 Dortmund, Germany

## Abstract

Aqueous Two-Phase Extraction is known to be a gentle separation technique for biochemical molecules where product partitioning is fast. However, the reason for the high mass transfer rates has not been investigated, yet. Many researchers claim that the low interfacial tension facilitates the formation of very small droplets and with it a large interfacial area causing a fast partitioning. However, an experimental evidence for this hypothesis has not been published yet. In this study, the mass transfer coefficients of two proteins, namely lysozyme and bromelain, were determined by providing a defined interfacial area for partitioning. Compared to low molecular weight solutes the mass transfer coefficient for the proteins investigated was small proving for the first time that the large interfacial area and not fast diffusion seems to be the reason for fast protein partitioning.

## Introduction

Aqueous Two-Phase Extraction (APTE) has been successfully applied as gentle unit operation for the purification of biomolecules such as therapeutic proteins^1–3^, enzymes^4^ and antibiotics^5,6^. Aqueous Two-Phase Systems (ATPSs) are known for fast product partitioning^7^, however, the reason for these high mass transfer rates has not been investigated so far. According to the kinetic two film model the rate of interphase mass transfer depends on the interfacial area as contact area between two liquid phases, the mass transfer coefficient, which is a measure of the specific mass transport rate across the interface, and the concentration difference, that is the driving force^8^. Cunha *et al*. briefly summarize, what Albertsson^9^, Husted *et al*.^10^ and Fauquex *et al*.^11^ already explained: “The fast approach to equilibrium is owing to the low interfacial tension between the two phases, which enables the formation of very small droplets and thus a large interface for mass transfer with low energy input”^12^. This assumption is shared by many researchers^12–19^. Although, the hypothesis is used over years, its evidence has not been proven so far.

In this study, the mass transfer coefficients of two macromolecules in an ATPS were determined by keeping the interfacial area constant during partitioning. That was realized by using a so-called Nitsch-Cell. This type of cell, first introduced by Lewis^20^, provides a known fixed interfacial area. The relatively simple set-up offers one of the most efficient methods to determine the mass transfer coefficient^8^. By determining the mass transfer coefficient and comparing it to values of other systems one can decide, whether the mass transfer is high due to a high mass transfer coefficient or due to the ability of the ATPS to be easily and efficiently dispersed generating a large interface.

## Results

**Figure 1:**
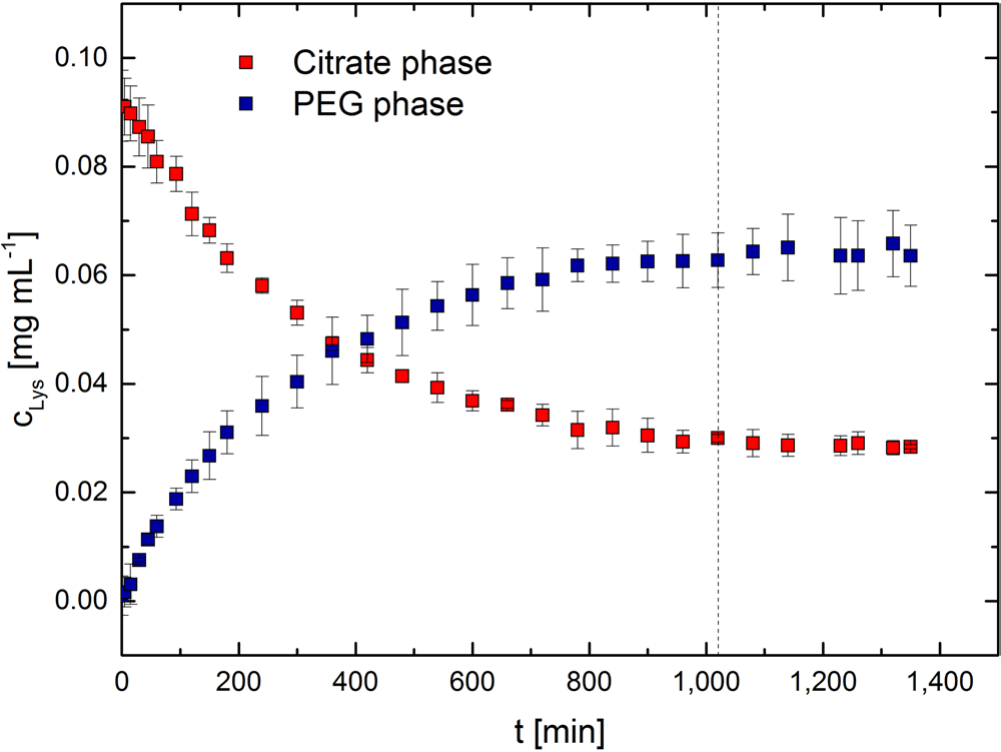
Concentration profile of lysozyme in the citrate and PEG phase over the time. As ATPS PEG4000/citrate with 2 wt.-% NaCl at 298.15 K was used. Profiles presented for a duplicate.

Concentration profiles of lysozyme and bromelain in the PEG and citrate phase were measured against time. The profiles for lysozyme in a PEG4000/citrate ATPS with 2 wt.-% NaCl are illustrated in Fig. 1. From the experiments it can be seen that the system reached equilibrium after about 1,020 min. For bromelain the equilibrium was reached after 1,080 min (see Supplementary Fig. S1). The mass balance was closed with an average accuracy of 2% for both systems.

**Figure 2:**
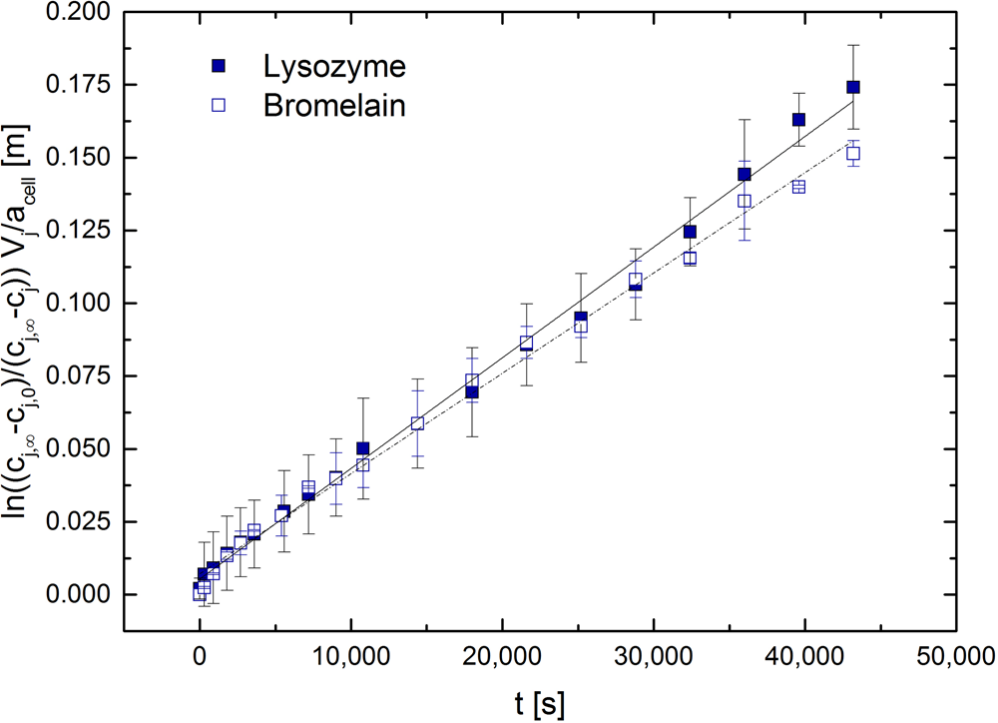
Determination of PEG mass transfer coefficients. Plot of $\mathrm{ln}\,\frac{({c}_{j,\infty }-{c}_{j,0})}{({c}_{j,\infty }-{c}_{j})}\cdot \frac{{V}_{j}}{{a}_{cell}}$ against time to obtain the PEG mass transfer coefficients of lysozyme and bromelain.

From the slope of the logarithmic profile of $\mathrm{ln}\,\frac{({c}_{j,\infty }-{c}_{j,0})}{({c}_{j,\infty }-{c}_{j})}\cdot \frac{{V}_{j}}{{a}_{cell}}$ plotted against time the PEG mass transfer coefficients were obtained as shown in Fig. 2. The time interval for the determination of *k* for both proteins was set to 720 min. The non-linear behaviour for bromelain in the beginning of the logarithmic profile might be a result of mixing the phases during the filling of the Nitsch-Cell. For lysozyme a PEG *k* -value of 3.79 × 10^−6^ m s^−1^ and for bromelain a PEG *k* -value of 3.44 × 10^−6^ m s^−1^ were determined by least square minimization. Mass transfer coefficients were plotted for the PEG phases only as the citrate *k* –value of lysozyme with 3.72 × 10^−6^ m s^−1^ was equal to the PEG one, which should be true for bromelain as well, once the mass balance is fulfilled.

## Discussion

Comparing the results with experiments obtained by Kruber *et al*. where the transfer of hexan between heptane and methanol was determined in the same Nitsch-Cell used here, the equilibrium was reached faster (approx. 60 min) and the *k* -value of 6.26 × 10^−5^ m s^−1^ ^21^ was 17–18 times higher than the ones measured in this study. Assuming an ideal mixing in each part of the compartment in the Nitsch-Cell, mass transfer is controlled by diffusion. As the mass transfer coefficient is related to the film thickness that is generally affected by the mixing conditions, higher stirrer speeds might lead to turbulent conditions and thus a thicker film. However, as the interface was stable due to the adjusted speed of the stirrer for the system in this study and for that examined by Kruber *et al*., it can be assumed that the significantly smaller mass transfer coefficients in this study cannot be related to the film thickness alone. The slower mass transfer obtained in this study might be influenced by the higher viscosity of the PEG phase, as with higher viscosity the diffusion coefficient decreases^22^. In addition, the molecular weight might affect the *k* -value, due to the increase in frictional resistance^23^. That is why further literature data was investigated: Kulaguin Chicaroux determined the concentration profile of the amino acid L-serine in a PEG8000/Dextran ATPS in the same Nitsch-Cell. The equilibrium was reached after approximately 300 min^24^. Comparing the value to the partitioning of lysozyme the equilibrium was reached approximately three times faster resulting in a *k* -value of 1.22 × 10^−5^ m s^−1^. Melgarejo-Torres measured for a cyclic ketone (M_W_ = 108.14 g mol^−1^) with a similar molecular weight compared to L-serine (M_W_ = 105.09 g mol^−1^) in a water ionic liquid biphasic system using a modified Lewis-Cell a *k*-value of 1.01 × 10^−5^ m s^−1^ ^25^. After comparing the physicochemical properties of the ionic liquid used in the study of Melgarejo-Torres, they differ in the viscosity of the PEG phase and interfacial tension to that of the ATPSs used in this study and to that of the PEG8000/Dextran system. The kinematic viscosity in the PEG phase used for lysozyme is significantly higher with a value of 23.86 mm^2^ s^−1^, measured by a capillary viscometer according to Kaplanow *et al*.^26^, compared to that of the ionic liquid with 2.5 × 10^−5^ mm^2^ s^−1^ ^25^. For the PEG8000/Dextran system the kinematic viscosity of the PEG phase is 8.6 mm^2^ s^−1^ and for the Dextran phase it is 44.9 mm^2^ s^−1^, both significantly higher in comparison to the water ionic liquid system^27^. Regarding the interfacial tension the one of the ionic liquid is with 7.5 mN m^−1^ about 60 times larger compared to the PEG/citrate ATPS without NaCl (0.125 mN m^−1^)^26^ and about 25 times larger compared to the same ATPS with exemplary 4 wt.-% NaCl (0.300 mN m^−1^). For the PEG8000/Dextran system a 125 times smaller interfacial tension of 0.060 mN m^−1^ was determined compared to the ionic liquid system^28^. Although the kinematic viscosity and the interfacial tension of the water ionic liquid system differs significantly to that of the PEG/Dextran system, the *k* -values determined were in the same range, as the molecular weight of the amino acid and the cyclic ketone were similar. However, for the PEG/citrate system the viscosity was approximately as high as for the PEG/Dextran APTS, but the molecular weight of lysozyme with 14.4 kDa^29^ is significantly higher compared to that of L-serine with 0.105 kDa. Thus, the small *k* -values for lysozyme and bromelain measured in the study were a result of a high molecular weight. Due to bromelain’s higher molecular weight of 21.4 kDa; measured via SDS-PAGE gel electrophoresis (own data), compared to lysozyme, it reached the equilibrium later than lysozyme. As the mass transfer in the Nitsch-Cell is controlled by diffusion only, the diffusion coefficients might give an explanation for the small mass transfer coefficients of macromolecules. Thereby, the diffusion coefficient is influenced by the solute size. The larger the molecular volume, the smaller the diffusion coefficient and the smaller the mass transfer coefficient.

The *k* -values for proteins investigated in this study are small. As the partitioning for proteins in an ATPS is fast, even at larger scale (30 L)^14^, the hypothesis seems to be true that the mass transfer is enhanced by generating large interfacial area only.

## Conclusion

In this study, the mass transfer coefficients of lysozyme and bromelain were determined for a constant interfacial area utilising a Nitsch-Cell. As the mass transfer rates of the proteins were 17 times lower compared to values of an organic compound in an organic/organic system, the fast equilibrium obtained in an ATPS seems to be caused by the large interfacial area generated during mixing. After comparing the physicochemical parameters of polymer/salt ATPS to those of polymer/polymer and water ionic liquid systems and the mass transfer coefficients of high and low molecular weight solutes, the molecular size seems to be the dominating parameter affecting the mass transfer coefficient.

## Materials and Methods

### Materials

Polyethylene glycol (PEG) with an average molecular weight of 4000 g mol^−1^ was obtained from Merck KGaA (Germany). Tri-sodiumcitrate dihydrate (Na_3_C_6_H_5_O_7_ * 2 H_2_O, ≥99.0 wt.-%) was purchased from VWR Chemicals (Belgium). Citric acid (C_6_H_8_O_7_, ≥99.0 wt.-%) and sodium chloride (NaCl, ≥99.8 wt.-%) were obtained from Carl Roth GmbH & Co. KG (Germany). The proteins lysozyme (from chicken egg white; ≥90.0 wt.-%) and bromelain (from pineapples, ≥40.0 wt.-%) were purchased from Sigma-Aldrich Co. (Germany). Pure deionized water was received from a Milli-Q Synthesis apparatus with 0.22 μm Millipack express filters from EMD Millipore Corporation (USA) and was used for all solutions throughout this work.

## Methods

### Preparation of ATPS

For all ATPS, PEG with a molecular weight of 4000 g mol^−1^, citrate as second phase forming component and NaCl as displacement agent to control partitioning were solved in deionized water. The pH value of 5.6 was adjusted by adding Na_3_C_6_H_5_O_7_ * 2 H_2_O and C_6_H_8_O_7_ as citrate components in a mass ratio of 13.2. The systems were weighted to reach 12.5 wt.-% of each phase forming compound. For lysozyme and bromelain an ATPS with 2 wt.-% and 6 wt.-% of NaCl, respectively, was prepared. All ATPSs were equilibrated and settled in a tempered double-walled dropping funnel at a constant temperature of 298.15 K. The equilibrated phases were separated and solutions were prepared for lysozyme (Lys) and bromelain (Bro) by dissolving 0.1 g_Lys_ L^−1^ or 1 g_Bro_ L^−1^ in the equilibrated citrate phase of an ATPS with 2 or 6 wt.-% NaCl, respectively. All solutions were stored at 298.15 K prior the experiments.

### Nitsch-Cell

**Figure 3:**
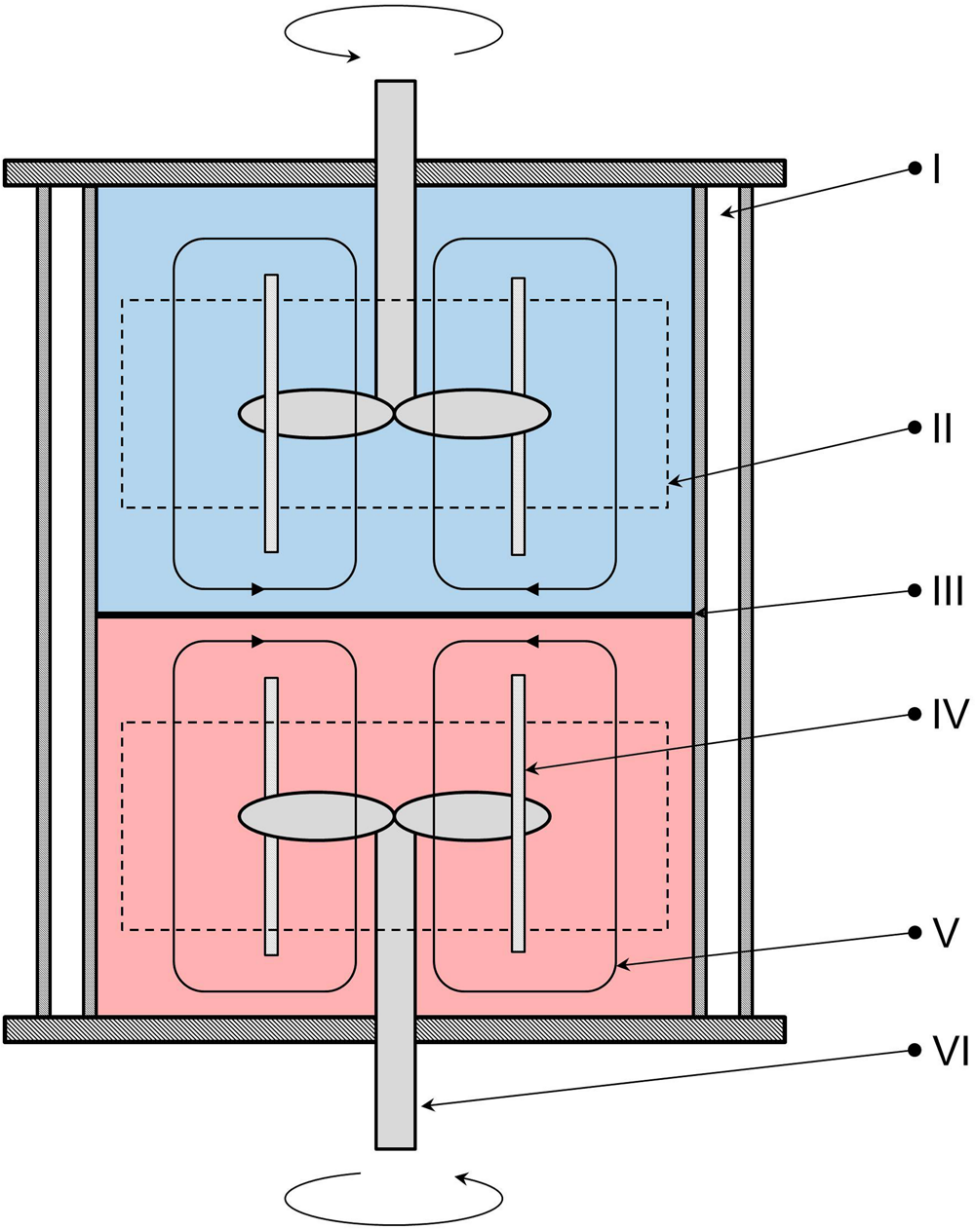
Schematic sketch of the Nitsch-Cell. (I) heating jacket; (II) flow tube; (III) interfacial area; (IV) baffle; (V) axial flow profile; (VI) integrated stirrer for lower phase.

A Nitsch-Cell^30^ consisting of a double-walled glass cylinder (I) with an internal diameter of 103.8 mm and a volume of 1.04 L was used to determine the mass transfer coefficients (Fig. 3). Two identical internal fittings consisting of a 28 mm flow tube (II) with an inner diameter of 72.5 mm and eight baffles (IV) with a height of 50 mm and integrated stirrer (VI) in both phases provided a convective flow from the interphase to the stirrer. To enable a stable interface with a constant area of 84.6 cm^2^, the stirrer speed was adjusted to keep the flow directions equal in both phases. Due to the higher viscosity in the PEG phase compared to the citrate phase, different agitation rates were adjusted. By stirring the PEG phase at 120 rpm and the citrate phase for 40 rpm a convective flow in each phase was provided and a stable interface was guaranteed. Silicone oil was used as heat transfer medium passing the double-walled jacket ensuring a constant temperature of 298.15 K. The experiments were performed as triplicates.

### Mass transfer coefficient experiments

The Nitsch-Cell was equally filled with citrate and PEG phase volume. After filling the Nitsch-Cell with citrate phase, the PEG phase was added carefully to prevent any mixing. The stirrers were started first with small agitation rates of 30 rpm for the citrate and 50 rpm for the PEG phase. After a clear interface became visible, the agitation rates were increased to 40 rpm and 120 rpm, respectively. In regular intervals samples of the citrate and PEG phase were taken for analysis using syringes.

The two-film theory is based on Fick’s first law and can be used to describe the mass transfer through a binary interface. Thereby, the assumption is the existence of stationary films on the two sides of the interface that are responsible for all the resistance to mass transfer and the mass transfer in the laminar interface layer is controlled by molecular diffusion. At the interface a linear equilibrium relationship between the PEG and the citrate phase and no concentration gradients in the bulk phases are assumed. As the mass transfer is purely diffusive, the film thickness might have an impact on the mass transfer coefficient. Thereby, the film thickness is related to the hydrodynamic properties, which depend on the average velocity of the liquid, the geometry and the physical properties of the fluids.

The mass transfer through the liquid/liquid interface is expressed by equation (1) for the phase *j* (here either PEG or citrate phase)^8^:

1 $${V}_{j}\cdot \frac{d{c}_{j}}{dt}={k}_{j}\cdot {a}_{cell}\cdot ({c}_{j,\infty }-{c}_{j})$$

where $\frac{d{c}_{j}}{dt}\,\,$is the mass transfer rate in the phase *j*, *V*_*j*_ is the compartment volume of the corresponding phase, *k*_*j*_ is the mass transfer coefficient, ${a}_{cell}$ is the interfacial area, ${c}_{j,\infty }$ is the final concentration in the bulk phase *j* (here the concentration reached at equilibrium), and *c*_*j*_ the concentration in the bulk phase *j* at time t. The equation (1) is applicable for the up taking side (here the PEG phase). The delivering side (here the citrate phase) has an opposite sign. Solving equation (1) at *t* = 0, *c*_*j*_ = *c*_*j*,*o*_, where *c*_*j*,*o*_ is the concentration in the beginning, results in equation (2):

2 $$\mathrm{ln}\,\frac{({c}_{j,\infty }-{c}_{j,0})}{({c}_{j,\infty }-{c}_{j})}\cdot \frac{{V}_{j}}{{a}_{cell}}={k}_{j}\cdot t$$

By plotting $\mathrm{ln}\,\frac{({c}_{j,\infty }-{c}_{j,0})}{({c}_{j,\infty }-{c}_{j})}\cdot \frac{{V}_{j}}{{a}_{cell}}$ against the time the mass transfer coefficient is equivalent to the slope of the curve.

### Analysis

Determination of lysozyme concentration was performed via high performance liquid chromatography (HPLC) using a Nucleodur^®^ C18EC column with a pore size of 300 Å and a particle size of 5 µm from Macherey-Nagel (Düren, Germany). The method described by Sutherland *et al*.^31^ was used with a modified gradient of the mobile phases (see Supplementary Table S1). The quantification of bromelain concentration was performed via UV absorption at 280 nm.

## Supplementary information

Mass Transfer of Aqueous Two-Phase Systems

## Data Availability

The data will be supplied upon request.
