# Supplementary material for: Mass Transfer of Proteins in Aqueous Two-Phase Systems
Source: Sci Rep. 2019 Mar 6;9:3692. doi: 10.1038/s41598-019-39797-9 (PMC6403220; doi:10.1038/s41598-019-39797-9)
Supplement: Supplementary file 1 — Mass Transfer of Aqueous Two-Phase Systems [file 41598_2019_39797_MOESM1_ESM.pdf]

Supplementary Information

Mass Transfer of Proteins in Aqueous Two-Phase Systems

I. Kaplanow, F. Goerzgen, J. Merz, G. Schembecker*

Laboratory for Plant and Process Design, Department of Biochemical and Chemical Engineering, TU Dortmund University, D-44227 Dortmund, Germany

Key words: Mass transfer coefficient, Aqueous Two-Phase Systems, Protein Partitioning, Nitsch-Cell

Supplementary data:

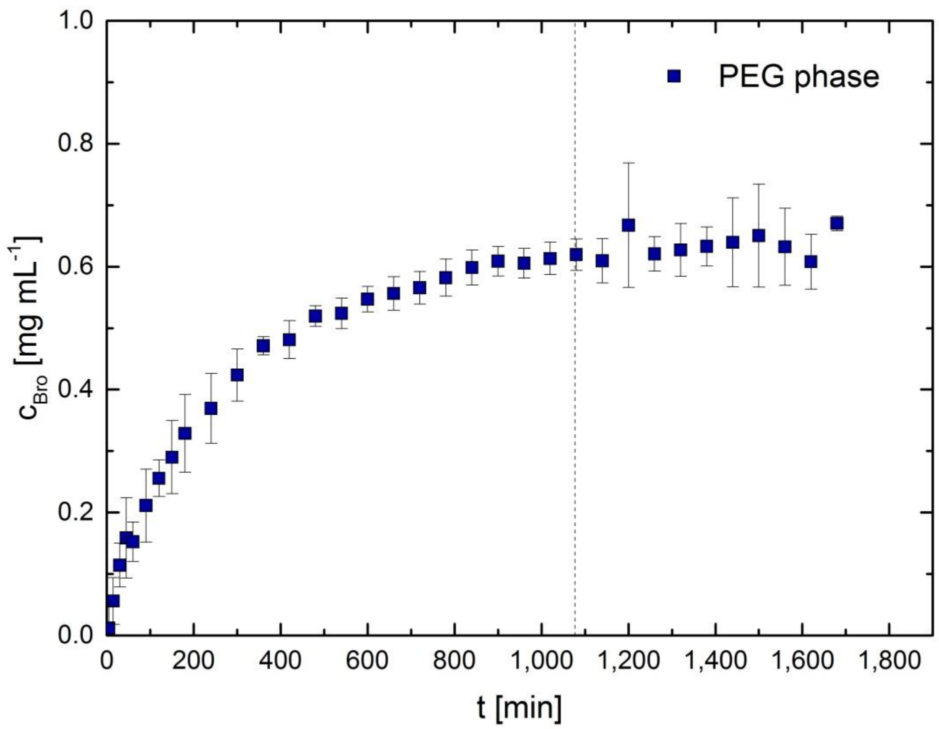

Figure S1: Concentration profile of bromelain in the PEG phase over the time.

As ATPS PEG4000/citrate with 6 wt.% NaCl at 298.15 K was used.

Table S1: HPLC method for Lys determination.

As column Nucleodur® C18EC at a flow rate of 1.5 mL/min and an injection volume of 20 µL were used. As mobile phase A acetonitrile with 0.05 Vol.% of triflouroacetic acid (TFA) and mobile phase B deionised water with 0.05 Vol.% TFA were used.

Time [min]	Vol.% A	Vol.% B
0	20	80
2	20	80
3	65	35
9	65	35
10	20	80
